# Supplementary material for: Immunocompetent cell targeting by food-additive titanium dioxide
Source: Nat Commun. 2025 Jul 4;16:6067. doi: 10.1038/s41467-025-60248-9 (PMC12227633; doi:10.1038/s41467-025-60248-9)
Supplement: Supplementary file 2 — Reporting Summary [file 41467_2025_60248_MOESM2_ESM.pdf]

nature portfolio

Corresponding author(s): Jonathan J. Powell

Last updated by author(s): Apr 6, 2025

Reporting Summary

Nature Portfolio wishes to improve the reproducibility of the work that we publish. This form provides structure for consistency and transparency in reporting. For further information on Nature Portfolio policies, see our Editorial Policies and the Editorial Policy Checklist.

Statistics

For all statistical analyses, confirm that the following items are present in the figure legend, table legend, main text, or Methods section.

n/a	Confirmed	
☐	☒	The exact sample size (*n*) for each experimental group/condition, given as a discrete number and unit of measurement
☐	☒	A statement on whether measurements were taken from distinct samples or whether the same sample was measured repeatedly
☐	☒	The statistical test(s) used AND whether they are one- or two-sided<br>*Only common tests should be described solely by name; describe more complex techniques in the Methods section.*
☒	☐	A description of all covariates tested
☐	☒	A description of any assumptions or corrections, such as tests of normality and adjustment for multiple comparisons
☐	☒	A full description of the statistical parameters including central tendency (e.g. means) or other basic estimates (e.g. regression coefficient) AND variation (e.g. standard deviation) or associated estimates of uncertainty (e.g. confidence intervals)
☐	☒	For null hypothesis testing, the test statistic (e.g. *F*, *t*, *r*) with confidence intervals, effect sizes, degrees of freedom and *P* value noted<br>*Give P values as exact values whenever suitable.*
☒	☐	For Bayesian analysis, information on the choice of priors and Markov chain Monte Carlo settings
☒	☐	For hierarchical and complex designs, identification of the appropriate level for tests and full reporting of outcomes
☒	☐	Estimates of effect sizes (e.g. Cohen's *d*, Pearson's *r*), indicating how they were calculated

Our web collection on statistics for biologists contains articles on many of the points above.

Software and code

Policy information about availability of computer code

Data collection

Confocal microscopy data was collected using Zeiss ZenBlack 2011 (version number 'SP2').

Transmission electron microscopy imaging and diffraction data were collected using Gatan's GMS v3.01.598.0 software with concordant energy dispersive X-ray analysis information collected using Thermofisher's Velox (version 2.9.0) software.

Scanning electron microscopy imaging data and energy dispersive X-ray data were collected were collected using ThermoFisher's Velox (version 2.9.0) software.

Inductively-coupled plasma mass spectrometry data was collected using Agilent's MassHunter 4.3 Workstation Software for 8900 ICP-QQQ, version C.01.03 Build 505.9 Patch 2.

Data analysis

With the exception of the proximity extension assay, all data analysis was conducted in MATLAB R2021b using the following toolboxes and version numbers:
MATLAB Version: 9.11.0.1809720 (R2021b) Update 1

Computer Vision Toolbox	Version 10.1	(R2021b)
Deep Learning Toolbox	Version 14.3	(R2021b)
Image Processing Toolbox	Version 11.4	(R2021b)
Parallel Computing Toolbox	Version 7.5	(R2021b)
Statistics and Machine Learning Toolbox	Version 12.2	(R2021b)

Single-cell feature extraction was performed using CellProfiler version 3.1.9
Image deconvolution was carried out using DeconvolutionLab_2 version 2.1.2 (27.06.2018)
Proximity extension assay data was analysed in the R programming environment (version 4.1.2) using the Olink Analyze package version 3.6.0.
All MATLAB code and sample data to reproduce our image analysis approach is available for download from the BioStudies database under accession number S-BSST875 (https://www.ebi.ac.uk/biostudies/bioimages/studies/S-BSST875)

For manuscripts utilizing custom algorithms or software that are central to the research but not yet described in published literature, software must be made available to editors and reviewers. We strongly encourage code deposition in a community repository (e.g. GitHub). See the Nature Portfolio guidelines for submitting code & software for further information.

Data

Policy information about availability of data

All manuscripts must include a data availability statement. This statement should provide the following information, where applicable:

- Accession codes, unique identifiers, or web links for publicly available datasets
- A description of any restrictions on data availability
- For clinical datasets or third party data, please ensure that the statement adheres to our policy

DATA AVAILABILITY
The data generated in this study have been deposited in the BioStudies database under accession code S-BSST875 [https://www.ebi.ac.uk/biostudies/bioimages/studies/S-BSST875]. All data underlying this study are available from the corresponding author upon request.

Research involving human participants, their data, or biological material

Policy information about studies with human participants or human data. See also policy information about sex, gender (identity/presentation), and sexual orientation and race, ethnicity and racism.

Reporting on sex and gender	Surgical specimens containing Peyer’s patches were collected from the neo-terminal ileum from six patients with Crohn’s disease or colonic cancer (3 males, 3 females; self-reported). These tissue samples were available for analysis with group-summary information only (i.e., resection date-range 2017-2018; median age 48 years; age-range 21 – 77 years) so disaggregation of the results in terms of sex was not possible. Alongside, three anonymised, formalin-fixed paraffin embedded (FFPE) human tissue samples containing ileal Peyer’s patch lymphoid tissue were also analysed to confirm if fgTiO2 could be detected in the FFPE specimen-type. Beyond tissue-type, no information about these human samples was available.
Reporting on race, ethnicity, or other socially relevant groupings	The six human tissue samples were sumarised in terms of male or female based on information from self-report. No other socially relevant groupings were used because the tissue samples were provided for analysis with group-summary information only (i.e., resection date-range 2017-2018; median age 48 years; age-range 21 – 77 years) and the goal was simply to determine if fgTiO2 was present in the Peyer's patch tissue regions or not.
Population characteristics	Resection date-range 2017-2018; median age 48 years; age-range 21 – 77 years.
Recruitment	Following written informed consent, and after approval from the Regional Ethical Review Board, Linköping, Sweden, human ileal samples were drawn at random from specimens collected from patients undergoing resection surgery for Crohn's or colonic cancer. In all instances tissue specimens formed the resection margins and were macroscopically normal. Microscopy analyses were carried out at Cambridge where the analysts were blinded to individual sample information and just provided with group-summary information (i.e., resection date-range 2017-2018; median age 48 years; age-range 21 – 77 years).
Ethics oversight	Studies using human tissues were approved by the Regional Ethical Review Board, Linköping, Sweden and by the UK NHS Health Research Authority, North West Greater Manchester East Research Ethics Committee, REC reference 18/NW/0690.

Note that full information on the approval of the study protocol must also be provided in the manuscript.

Field-specific reporting

Please select the one below that is the best fit for your research. If you are not sure, read the appropriate sections before making your selection.

[x] Life sciences [ ] Behavioural & social sciences [ ] Ecological, evolutionary & environmental sciences

For a reference copy of the document with all sections, see nature.com/documents/nr-reporting-summary-flat.pdf

Life sciences study design

All studies must disclose on these points even when the disclosure is negative.

Sample size	Sample sizes (i.e., n = 3-6 mice per treatment group) were determined based on experience from our previous work rather than by statistical method. The nature of our high-throughput imaging approach allows statistically defensible populations containing more than 10^4 cells to be analysed from each tissue section. This - alongside the ability to target precise tissue regions - provided excellent sensitivity to detect subtle effects between treatment groups whilst minimising the total numbers of animals required.

Data exclusions	As is standard practice for image-based cell profiling, cell-objects with areas outside of the 5th-to-95% percentile were discarded prior to analysis to minimise the impact of any mis-segmented cells on the results obtained. Similarly, any cell-objects partially obscured due to overlapping the edges of the imaging field-of-view were also discarded to ensure per-cell information was accurately quantified.
Replication	The presented work describes data collected from n =3-6 mice per treatment group and timepoint repeated across two independent mouse studies and n = 9 human individuals. Each result was validated by replication across at least three tissue sections per individual.
Randomization	In the two mouse studies, six-week-old mice were randomly allocated into the respective treatment groups at the beginning of the study. For the human studies to check for the presence of fgTiO2 in Peyer's patches, three male and three female frozen human samples and three anonymised parafin-embedded tissue samples were randomly drawn from available tissue holdings. During imaging experiments, slide-mounted tissue sections per diet-group were randomly allocated to different antibody staining experiments.
Blinding	Investigators were blinded to mouse diet-group allocation during data collection. However, investigators were not blinded to sample identities during analysis because it was necessary to know which mouse tissues were exposed to fgTiO2 in order to set up the image analysis pipelines (i.e., set thresholds for the detection of fgTiO2 or immuno-histochemical markers from background as described in Supplementary Figure 1). However image data were then processed automatically by computer measurement and not scored by humans with prior knowledge of the sample identities - so this knowledge had no impact on the tissue measurements taken.

Reporting for specific materials, systems and methods

We require information from authors about some types of materials, experimental systems and methods used in many studies. Here, indicate whether each material, system or method listed is relevant to your study. If you are not sure if a list item applies to your research, read the appropriate section before selecting a response.

Materials & experimental systems

n/a	Involved in the study	
☐	☒	Antibodies
☒	☐	Eukaryotic cell lines
☒	☐	Palaeontology and archaeology
☐	☒	Animals and other organisms
☒	☐	Clinical data
☒	☐	Dual use research of concern
☒	☐	Plants

Methods

n/a	Involved in the study	
☒	☐	ChIP-seq
☒	☐	Flow cytometry
☒	☐	MRI-based neuroimaging

Antibodies

Antibodies used

Supplementary Table 2 in the manuscript describes the antibodies used in the study in full - including manufacturer, product number, clone, dilutions, stock concentrations, host animal, and the secondary antibody / fluorophore used for detection for each Figure-component. In brief:

NAME - PRODUCT NO - CLONE - SUPPLIER - DILUTION - STOCK CONCENTRATION - - HOST:
Anti-mouse GP2 - D278-3 - 2F11-C3 - MBL - 1:200 - 1mg/mL -- Rat
Anti-mouse CD3 - AB5690 - polyclonal - ABCAM - 1:150 - 0.2 mg/mL -- Rabbit
Anti-mouse CD11c - AB33483 - N418 - ABCAM - 1:400 - 0.5 mg/mL -- Armenian Hamster
Anti-mouse B220 - 50045280 - RA3-6B2 - ThermoFisher - 1:50 - 0.2mg/mL -- Rat
Anti-mouse CD11c - 53011482 - N418 - ThermoFisher - 1:25 - 0.2mg/mL -- Armenian Hamster
Anti-mouse CD3 - 17003282 - 17A2 - ThermoFisher - 1:50 - 0.2 mg/mL -- Rat
Anti-mouse PD-L1 - AB213480 - EPR20529 - ABCAM - 1:100 - 0.5 mg/mL -- Rabbit
Anti-mouse MHCII - PE-65122-100UG - m5/114.15.2 - Thermo-Fisher - 1:25 - 0.2mg/mL -- Rat
Anti-mouse CD4 - 12-0041-82 - GK1.5 - ThermoFisher - 1:25 - 0.2 mg/mL -- Rat
Anti-flagellin - AB93713 - polyclonal - Abcam - 1:100 - 1mg/mL -- Rabbit

Validation

All immunofluorescence experiments used concentration-matched isotype controls to assess non-specific antibody binding. These distributions are presented in Figure 4e/f/g (B220,CD3,CD11c) Figure 4k (CD11c) and Figure 4w / Supplementary Figure 10 (PD-L1). Common cell phenotypic markers (i.e., GP2 / CD3 / CD11c / B220 / CD4 / MHCII) were further validated in mouse tissues using established knowledge of cell locations in intestinal Peyer's patches. GP2 signal delineating microfold (M) cells was confirmed present in the M-cell rich, follicle associated epithelium overlying Peyer's patches whilst absent in the regular epithelium overlying the lamina propria (shown, Figure 2k). CD3 and B220 signal (delineating T and B lymphocytes, respectively) strongly associated with Peyer's patch T-cell zones and germinal centers (respectively) (shown, 4a/b and Figure 2k). CD4 labelled Peyer's patch T-cell zones as expected (shown, Supplementary Figure 14). MHCII showed pronounced staining throughout the Peyer's patch as is expected in a 'B-cell follicle' (shown, Supplementary Figure 14). CD11c / signal delineating mononuclear phagocyte cells was enriched in the subepithelial dome region of the Murine Peyer's patch - as expected (shown, Figure 4i). In addition to secondary-only and isotype controls (shown, Supplementary Figure 10) the PD-L1 antibody is recombinant and monoclonal with knock-out validation and western blot size confirmation (single ~50kDa band) data available at https://www.abcam.com/pd-l1-antibody-epr20529-ab213480.html The anti-flagellin staining for Salmonella was validated using biological positive control tissues (i.e., mouse Peyer's patch tissue known to contain Salmonella, data shown Figure 5a-c).

Animals and other research organisms

Policy information about studies involving animals; ARRIVE guidelines recommended for reporting animal research, and Sex and Gender in Research

Laboratory animals	'Mouse study 1' used 48, six-week-old mice (Mus musculus, C57BL/6J, 50:50 male:female) (Source, AgResearch Ruakura Small Animal Colony). 'Mouse study 2' used 36, six-week-old mice (Mus musculus, C57BL/6NCrl, female) (Source, Charles-River Laboratories).
Wild animals	This study did not use wild animals.
Reporting on sex	In previous work using tissues from the same animals we extensively characterised for sex-specific differences in terms of TiO2 intake and delivery to small intestinal Peyer's patches. No statistically significant sex-specific differences were determined (https://doi.org/10.1002/smll.202000486). For these reasons and to maximize statistical power, samples from males and females were analysed togther in the presented work. During anlaysis, information describing the sex of individual tissue sections was not retained so the presented data cannot be disaggregated by sex.
Field-collected samples	This study did not use samples collected from the field.
Ethics oversight	The mouse fgTiO2 feeding study was approved by the Grasslands Animal Ethics Committee (Palmerston North, New Zealand) in accordance with the New Zealand Animal Welfare Act 1999. Studies at Cambridge were also approved by the UK NHS Health Research Authority, North West – Greater Manchester East Research Ethics Committee, REC reference 18/NW/0690.

Note that full information on the approval of the study protocol must also be provided in the manuscript.

Plants

Seed stocks	NA
Novel plant genotypes	NA
Authentication	NA
