# Supplementary material for: Immunocompetent cell targeting by food-additive titanium dioxide
Source: Nat Commun. 2025 Jul 4;16:6067. doi: 10.1038/s41467-025-60248-9 (PMC12227633; doi:10.1038/s41467-025-60248-9)
Supplement: Supplementary file 3 — Description of Additional Supplementary Files [file 41467_2025_60248_MOESM3_ESM.pdf]

Supplementary Movie 1 – 3-D animation demonstrating the selectivity and specificity of fgTiO_2 for autofluorescent LysoMac / LysoDC immune cells. The video shows a 3-D render of image-data collected as a Z-stack from the murine subepithelial dome tissue compartment using confocal reflectance microscopy. Cell nuclei were fluorescently labelled using Hoechst 33342 (grey). Cytoskeletal actin was labelled with phalloidin-AlexaFluor 647 (red). fgTiO_2 (white foci) are seen to selectively and specifically target the highly autofluorescent (blue) mononuclear phagocytic cells.
